# Isovitexin Alleviates Myocardial Ischemia by Targeting SLC25A4 and Modulating the AMPK/PGC-1*α* Signaling Pathway

**DOI:** 10.3390/ijms27104193

**Published:** 2026-05-08

**Authors:** Zilu He, Zaozhen Shan, Yi Zhou

**Affiliations:** 1 College of Eco-Environmental Engineering, Qinghai University, Xining 810016, China; ys230901j10560@qhu.edu.cn (Z.H.); 15911428923@139.com (Z.S.); 2 School of Pharmacy, Qinghai University, Xining 810016, China

**Keywords:** ISOV, myocardial ischemia, SLC25A4, AMPK/PGC-1α, mitochondrial function

## Abstract

Isovitexin (ISOV) is an active component identified in the traditional Tibetan medicine Tsantan Sumtang, which is commonly used for treating myocardial ischemia. Although previous studies have suggested the protective effect of ISOV on cardiomyocytes, the in vivo anti-ischemic efficacy and underlying mechanisms of ISOV remain unclear. This study aimed to systematically evaluate the therapeutic effects of ISOV on myocardial ischemia in rats and to elucidate its molecular mechanism of action. An acute myocardial infarction model was established in rats by ligating the left anterior descending branch (LADL) of the coronary artery. The protective effects of ISOV were assessed by measuring infarct size, serum cardiac injury biomarkers, and oxidative stress levels. Chemical proteomics using photoaffinity magnetic beads was employed to identify potential target proteins of ISOV. Molecular docking, pull-down western blotting, and cellular thermal shift assay (CETSA) western blotting were applied to validate the interaction between ISOV and target. Knockdown of the target was used to verify the mechanism of ISOV on anti-myocardial ischemia effect. ISOV treatment significantly reduced myocardial infarct size, decreased serum levels of lactate dehydrogenase (LDH), creatine kinase isoenzymes (CK-MB), malondialdehyde (MDA), and enhanced superoxide dismutase (SOD) activity in myocardial ischemia rats. Furthermore, ISOV improved mitochondrial function, as evidenced by increased ATP content and enhanced activities of mitochondrial complexes I and IV. Chemical proteomics and bioinformatic analysis identified SLC25A4 as a direct target of ISOV. Molecular docking revealed a high-affinity binding (binding energy: −8.3 kcal/mol), which was further confirmed by pull-down assays and CETSA. In SLC25A4-knockdown H9c2 cells under hypoxic conditions, ISOV upregulated SLC25A4 expression, promoted the phosphorylation of adenosine monophosphate (AMP)-activated protein kinase (AMPK) and upregulated the expression of proliferator-activated receptor gamma coactivator-1α (PGC-1α). ISOV exerts cardioprotective effects against myocardial ischemia by directly binding to SLC25A4 and activating the AMPK/PGC-1α pathway, highlighting its potential as a therapeutic agent for myocardial ischemia.

## 1. Introduction

Myocardial ischemia is a major cause of death worldwide [1] its core pathological feature is the imbalance between myocardial oxygen supply and demand caused by coronary artery and microcirculation disorders [2]. Although revascularization is the main clinical treatment method, the reperfusion process may cause additional myocardial damage [3], Recent studies have found that mitochondrial dysfunction occurs in the myocardial ischemia process [4]. Therefore, regulation of mitochondrial structure and function to maintain myocardial energy homeostasis has become an important research direction in the field of myocardial ischemia treatment [5,6].

The cardiac mitochondria generate ATP through oxidative phosphorylation. Myocardial ischemia induces ischemia and hypoxia, which affects this process. Reduced ATP production leads to energy disorders [7], disruption of mitochondrial calcium homeostasis causes calcium overload, resulting in a decrease in mitochondrial membrane potential (MMP), opening of the Mitochondrial Permeability Transition Pore (mPTP), and triggering cell apoptosis [8], increased oxidative stress leads to free radical damage to mitochondria [9], myocardial ischemia also disrupts the quality control of mitochondria, causing the accumulation of damaged mitochondria and affecting myocardial function [10]. These changes collectively exacerbate the damage process of myocardial cells.

**Figure 1:**
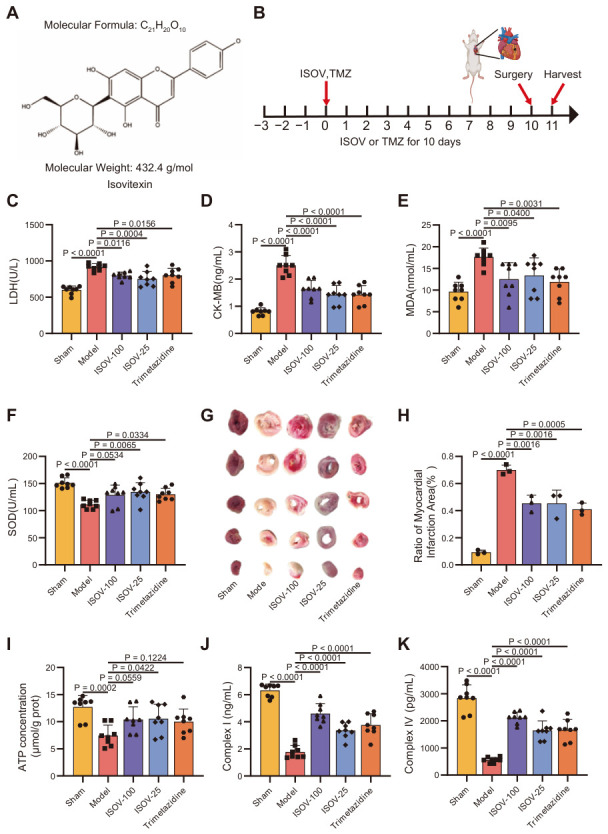
ISOV protects rats from MI injury. (**A**) The structural formula of ISOV. (**B**) Schematic diagram of the experimental time for the LADL model animals: ISOV (25 and 100 mg/kg/day, ig) was continuously administered for 10 days, and the surgery was performed on the 10th day of treatment, followed by the sacrifice of rats 24 h after the operation. (**C**–**F**) Serum LDH, CK-MB, MDA and total SOD activity in each group of LADL model rats (n = 8). (**G**,**H**) Representative images of TTC and calculated myocardial infarction area (n = 8). (**I**–**K**) ATP, Complex I, and Complex IV in the heart tissue of each group of LADL model rats (n = 8). Data are expressed as mean ± SEM.

Isovitexin (Figure 1A), an active compound identified by our research team in an earlier study from the classic Tibetan medicine Tsantan Sumtang (used for treating myocardial ischemia), is a naturally occurring flavonoid. ISOV exhibits a wide range of pharmacological activities, including antioxidant, anti-inflammatory, anticancer, cardioprotective, and hepatoprotective effects [11,12,13]. Previous studies have shown that ISOV can significantly improve oxidative stress and energy metabolism disorders in the H9c2 cell model with hypoxic injury [14]. In addition, ISOV has been shown to exert protective effects by regulating mitochondrial function in various disease models, such as promoting osteogenic differentiation [15] and improving renal injury [16]. These findings collectively highlight the diverse pharmacological potential of ISOV [17].

Although our earlier findings suggested that ISOV may mitigate myocardial ischemia through mitochondrial protection, its specific molecular target and detailed mechanism remain unclear. Therefore, this study integrates pharmacodynamic evaluation, chemical proteomics, pull-down-WB, cellular thermal shift assay (CETSA)-WB, molecular docking, and gene knockdown techniques to systematically explore the pharmacodynamic effect and molecular mechanism of ISOV on counteracting myocardial ischemia. The research was conducted using both animal models and cell-based assays to comprehensively determine how ISOV exerts its anti-ischemic effects.

## 2. Results

### 2.1. ISOV Can Improve Myocardial Ischemic Injury in Rats

To evaluate the cardioprotective effects of ISOV, we established a rat myocardial infarction model. Figure 1B shows the schematic diagram of the animal experiment for the myocardial infarction model group. ISOV pretreatment at both 25 and 100 mg/kg significantly reduced serum LDH and CK-MB levels (*p* < 0.01, Figure 1C,D), with little difference between the two doses. ISOV also alleviated oxidative stress, as evidenced by a significant decrease in MDA content (*p* < 0.05 for 25 mg/kg, *p* < 0.01 for 100 mg/kg, Figure 1E), while SOD activity showed an upward trend without statistical significance (Figure 1F). TTC staining revealed that ISOV at both doses significantly reduced the myocardial infarction area (*p* < 0.01, Figure 1G,H), with the higher dose showing a more pronounced effect. Given that mitochondrial dysfunction (characterized by impaired respiratory chain activity and insufficient ATP production) is an important pathogenesis of cardiovascular diseases [18,19], we further assessed mitochondrial function. ISOV treatment (25 and 100 mg/kg) significantly enhanced ATP production and increased the activities of mitochondrial respiratory chain complexes I and IV, with the higher dose again showing a more pronounced effect (*p* < 0.01, Figure 1I–K). These results indicate that ISOV confers dose dependent protection against myocardial infarction by preserving mitochondrial energy metabolism.

### 2.2. Chemical Proteomics Utilizing Photoaffinity Probe-Based Pull-Down Assays Identified Potential Targets of ISOV in H9c2 Cells

**Figure 2:**
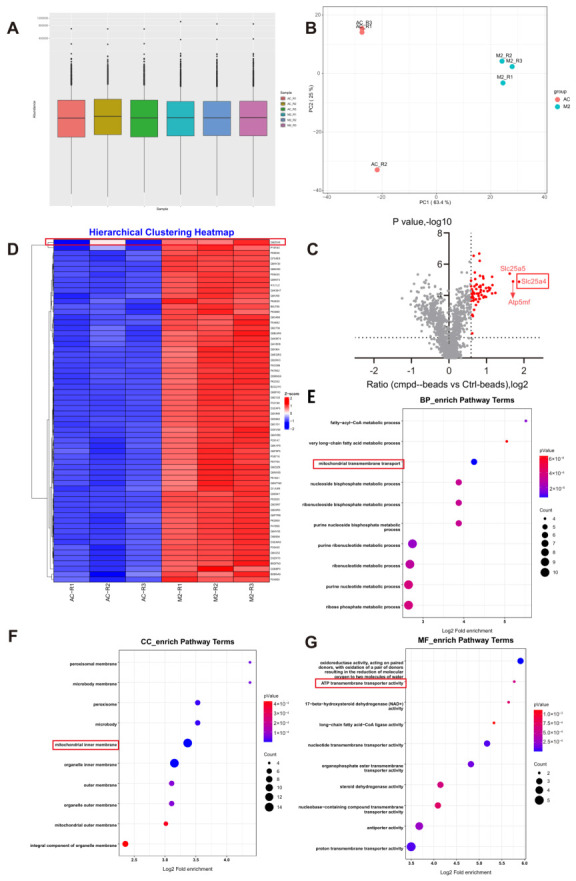
Targeting in Chemical Proteomics Technology. (**A**) Quantitative protein box plot. (**B**) PCA component analysis diagram. (**C**) Targeting volcano plot. (**D**) Hierarchical clustering heatmap of proteins enriched in the ISOV probe (M2) versus control bead (AC) pull-down experiments. (**E**–**G**) Bar charts showing the enrichment analysis of GO pathways for differentially expressed proteins in the M2 group and the AC group. (Mean ± SEM, n = 3).

To identify the direct cellular targets of ISOV in H9c2 cardiomyocytes, we employed a photoaffinity probe-based pull-down strategy followed by LC-MS/MS analysis. A total of 245,842 MS/MS spectra were acquired, of which 28,924 were successfully matched to the database, corresponding to 11,517 unique peptides. Although quantitative proteomic profiling showed comparable overall protein abundance between the ISOV-treated (M2) and control (AC) groups (Figure 2A), principal component analysis (PCA) revealed clear separation along the first principal component (PC1), suggesting substantial differences in protein expression patterns between conditions (Figure 2B). Using a threshold of |Abundance ratio| > 1.5 and *p* < 0.05, we identified 64 differentially expressed proteins (DEPs), which were prominently highlighted in the volcano plot (Figure 2C). Among these, SLC25A4 (also known as mitochondrial adenosine monophosphate transporter 4) emerged as the most significantly upregulated protein in the M2 group (Abundance ratio = 3.638, *p* = 0.0000134).

Bioinformatic analysis further demonstrated that the DEPs were predominantly associated with mitochondrial function. Gene Ontology (GO) enrichment analysis indicated significant enrichment in biological processes related to mitochondrial transmembrane transport (Figure 2D), cellular components including the inner mitochondrial membrane (Figure 2E), and molecular functions such as ATP transmembrane transporter activity (Figure 2F). Integrative analysis combining chemical proteomics and network pharmacology suggested that ISOV likely targets the mitochondrial transporter SLC25A4, modulating mitochondrial energy metabolism and thereby contributing to its anti-myocardial ischemic effects.

### 2.3. SLC25a4 Is a Direct Binding Target in H9c2 Cells

**Figure 3:**
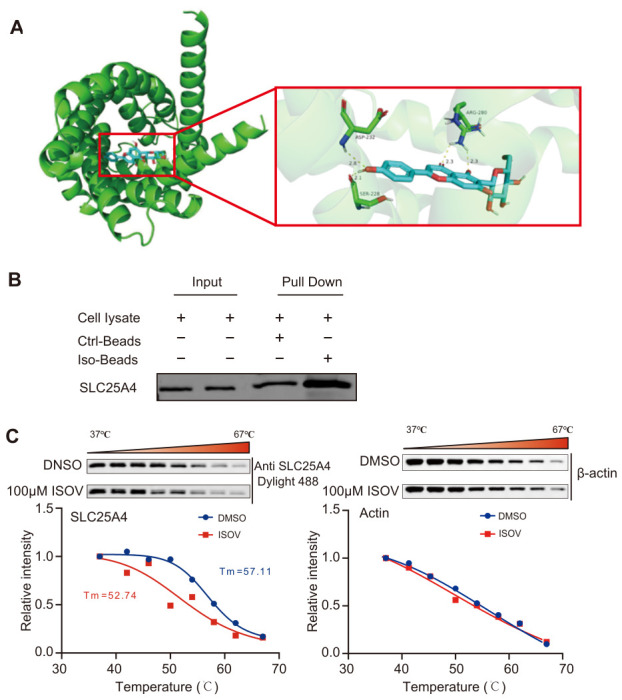
SLC25a4 is a direct binding target in H9c2 cells. (**A**) Molecular docking model of the binding of ISOV to SLC25A4. (**B**) Verification of the binding of ISOV to SLC25A4 by immunoprecipitation followed by Western blotting. (**C**) Verification of the binding of ISOV to SLC25A4 by CETSA (n = 3).

Through molecular docking simulations, it was found that the binding energy of ISOV to SLC25A4 was −8.3 kcal/mol (Figure 3A), indicating a strong binding affinity between the two. After the pull-down assay, Western blotting analysis confirmed that the functionalized magnetic beads of ISOV could specifically capture the SLC25A4 protein (Figure 3B). Further CETSA was conducted, and ISOV treatment significantly reduced the thermal stability of SLC25A4 (Figure 3C). This study reveals that ISOV can directly target the SLC25A4 protein in hypoxia-induced H9c2 cells, providing an important molecular basis for elucidating the mechanism of ISOV’s cardioprotective effect.

### 2.4. ISOV Activates the SLC25A4 Signaling Pathway, Thereby Mitigating Hypoxia-Induced Oxidative Stress, Myocardial Injury, and Mitochondrial Dysfunction in H9c2 Cells

**Figure 4:**
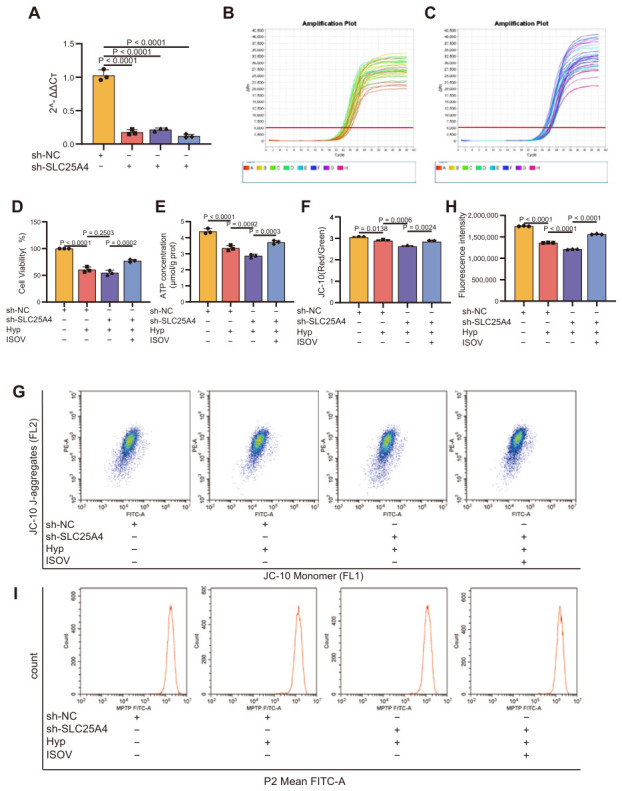
The effects of SLC25A4 knockdown and ISOV intervention on mitochondrial function and cell viability of H9c2 cells under hypoxia. (**A**–**C**) Verification of SLC25A4 knockdown efficiency. (**D**,**E**) Detection of cell viability (CCK8 method) and ATP content (fluorescence method). (**F**,**G**) MMP (JC-1 probe detection). (**H**,**I**) Quantitative results of mPTP opening rate (fluorescence polarization method) (Mean ± SEM, n = 3).

To investigate whether ISOV exerts anti-myocardial ischemia effects by targeting the SLC25A4-dependent signaling pathway, we constructed a SLC25A4 knockdown model in hypoxia-induced rat H9c2 cardiomyocytes using shRNA. To investigate the functional role of SLC25A4, we established a stable knockdown model in H9c2 cells using shRNA. The knockdown efficiency was first confirmed at the transcriptional level by qPCR, which showed a significant decrease in SLC25A4 mRNA expression (Figure 4A–C). The experimental results showed that SLC25A4 knockdown significantly inhibited the viability of H9c2 cells under hypoxia stress (Figure 4D), reduced intracellular ATP levels (Figure 4E), and caused a significant decrease in MMP (Figure 4F,G), and accelerated the opening of the mPTP (Figure 4H,I). Notably, after ISOV intervention, the above pathological manifestations were significantly improved. The cell survival rate in the ISOV treatment group significantly increased, ATP content significantly increased, MMP significantly recovered, and the mPTP opening rate decreased. These research results suggest that the protective effect of ISOV on hypoxia injury may be directly related to the regulation of SLC25A4 function.

**Figure 5:**
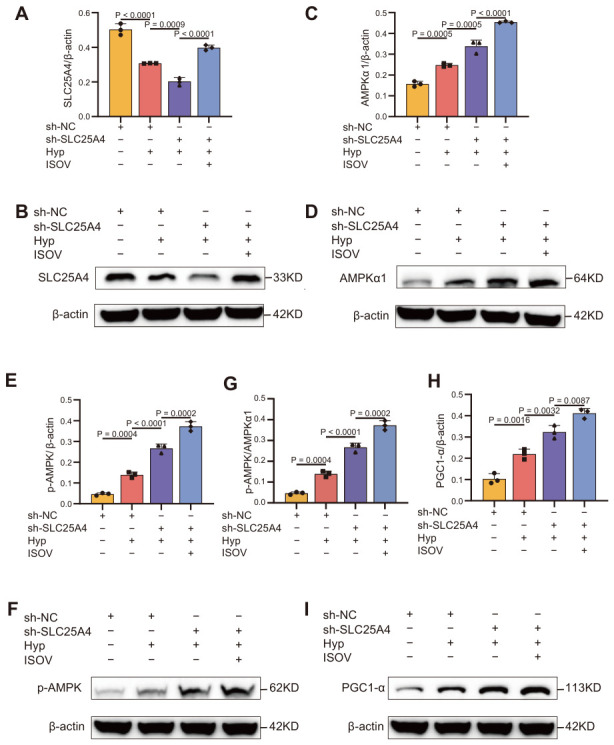
Effects of SLC25A4 knockdown and ISOV intervention on the AMPK/PGC-1α signaling pathway under hypoxic conditions. (**A**,**C**,**E**,**H**) Corresponding semi-quantitative analyses. (**G**) Semi-quantitative analysis of the p-AMPK/AMPKα1 ratio. (**B**,**D**,**F**,**I**) Representative Western blot bands for SLC25A4, AMPKα, p-AMPK, and PGC-1α, respectively. Data are presented as mean ± SEM (n = 3).

To further investigate the mechanism of action of ISOV, we analyzed the protein expression changes in key metabolic regulatory pathways by Western blotting. Knockdown of SLC25A4 markedly reduced the expression of endogenous SLC25A4 protein (Figure 5A,B). In contrast, ISOV treatment significantly up-regulated its expression (*p* < 0.01), restoring SLC25A4 protein levels to approximately 79% of baseline. Under hypoxia stress conditions, the expression of AMP-activated protein kinase α (AMPKα) and its phosphorylated form (p-AMPK) in the SLC25A4 knockdown group significantly increased (Figure 5C–F). Moreover, ISOV treatment significantly increased the p AMPK/AMPKα1 ratio under hypoxia compared to the hypoxia group (*p* < 0.05, Figure 5G). Additionally, the expression of peroxisome proliferator-activated receptor γ coactivator 1-α (PGC1-α) also showed a significant upward trend (Figure 5H,I). When ISOV was combined with SLC25A4 knockdown treatment, the expression levels of AMPKα, p-AMPK, and PGC1-α further significantly increased (Figure 5C–I). This indicates that in the SLC25A4-deficient cell model, ISOV significantly enhanced the activity of the AMPK-PGC1-α signaling pathway, effectively alleviating mitochondrial dysfunction, providing a clear molecular mechanism explanation for the mitochondrial protective effect of ISOV.

## 3. Discussion

This study provides the first evidence that ISOV, a bioactive compound derived from the traditional Tibetan medicine Tsantan Sumtang, exerts significant cardioprotective effects against myocardial ischemia by targeting SLC25A4/ANT1 and activating the AMPK/PGC-1α signaling axis. Myocardial ischemia, a leading cause of mortality worldwide [20,21], is characterized by impaired coronary blood flow resulting in disrupted energy metabolism and cardiomyocyte death [22]. In this study, ISOV treatment markedly reduced infarct size in animal models, an effect closely associated with the preservation of mitochondrial function. We acknowledge that our pretreatment protocol does not reflect clinical practice. Future studies should evaluate ISOV administered after ischemia injury.

Using a Photoaffinity Probe-Based Pull-Down Assays coupled with LC-MS/MS analysis [23], we identified 64 potential protein targets in H9c2 cardiomyocytes, among which SLC25A4 was selected as the highest-confidence direct binding target. SLC25A4 is a key transporter located in the inner mitochondrial membrane responsible for ADP/ATP exchange, playing a central role in cellular energy homeostasis [24]. It also regulates the opening of the mPTP [25,26], thereby serving as a critical node linking energy metabolism and apoptotic signaling [27]. It is highly expressed in energy-demanding tissues such as heart and skeletal muscle [28]. Subsequently, we constructed a complete evidence chain through molecular docking (binding energy −8.3 kcal/mol), Pull-down western blotting, and CETSA western blotting, confirming the direct binding between ISOV and SLC25A4. In the CETSA experiment, the ISOV treatment group showed a significant decrease in thermal stability, contrasting with the thermal stabilization typically observed upon ligand binding. This difference might be explained by a conformational change in SLC25A4 induced by ISOV binding, making the protein more sensitive to heat-induced denaturation and aggregation. Importantly, the thermal destabilization observed after ISOV treatment, like thermal stabilization, constitutes reliable evidence for a direct interaction between the protein and the ligand [29]. This binding may regulate the transport activity of SLC25A4, affecting the intracellular AMP/ATP balance, thereby activating Adenosine monophosphate (AMP)–activated protein kinase (AMPK) and its downstream signaling. Activated AMPK further upregulates its key downstream effector molecule proliferator-activated receptor gamma coactivator-1α (PGC-1α) [30].

In the functional validation experiment, knockdown of SLC25A4 resulted in decreased ATP levels, reduced MMP, and opening of the mPTP. In contrast, ISOV treatment upregulates SLC25A4 expression to approximately 70%, thereby restoring its function. Consequently, after ISOV intervention, ATP levels and MMP were elevated, and mPTP opening was suppressed. Western blotting analysis revealed that ISOV significantly enhanced AMPK phosphorylation. AMPK acts as a central cellular energy sensor that responds to changes in the AMP/ATP ratio and orchestrates metabolic adaptation to stress [31]. AMPK is a key metabolic regulator that senses energy status and controls energy expenditure and storage. AMPK is allosterically activated by AMP and repressed by adenosine triphosphate (ATP) [32]. Activated AMPK subsequently upregulates its key downstream effector, PGC-1α, a master regulator of mitochondrial biogenesis. The peroxisome PGC-1α is a key regulatory factor for mitochondrial generation [33].

Downregulation or transcriptional blockade of PGC-1α has been observed in heart failure and myocardial infarction models [34,35]. Compelling evidence indicates that AMPK activation improves cardiac contractility post-infarction by enhancing PGC-1α expression and mitochondrial respiration [36]. As the main regulatory factor for mitochondrial biogenesis, the increased expression of PGC-1α promotes the generation, repair and functional optimization of mitochondria, thereby enhancing the energy supply capacity and antioxidant stress resistance of cardiomyocytes under ischemic stress, and ultimately achieving the protective effect on cardiomyocytes. Moreover, activation of the AMPK/PGC-1α pathway attenuates mitochondrial dysfunction, suppresses apoptosis [37], and counteracts acute cardiotoxicity by promoting mitochondrial homeostasis [38]. These findings suggest that this pathway may represent a potential therapeutic target for ischemic heart disease. Our in vitro findings demonstrate that ISOV regulates the SLC25A4/AMPK/PGC-1α pathway in H9c2 cells. Nevertheless, confirmation in animal models is necessary to validate its translational relevance, a goal for future studies.Importantly, although ISOV enhanced AMPK/PGC-1α expression via SLC25A4, it partially activated the pathway even in SLC25A4-knockdown cells, suggesting a multi-target mechanism of action and underscoring the pivotal role of SLC25A4 within the cellular energy network.

## 4. Materials and Methods

### 4.1. Chemical and Reagents

The ISOV was obtained from Northern Weiye Metrology Group Co., Ltd. (Zhengzhou, Henan, China). Detailed information on other chemicals and reagents is provided in the in the Supplementary Materials (Table S1).

### 4.2. Animal

Forty specific-pathogen-free (SPF) 12-week-old healthy male Sprague-Dawley (SD) rats (weighing 250–300 g) were purchased from Himalaya Animal Experiment Center Co., Ltd. (Xining, Qinghai, China; license number: SCXK(Qing) 2024-0001).

Forty SD rats were randomly assigned to five groups (n = 8 per group): sham, model, trimetazidine (6.3 mg/kg/day, i.g.)( trimetazidine purchased from Servier, Suresnes, France), ISOV (25 mg/kg/day, i.g.), and ISOV (100 mg/kg/day, i.g.). Each group was administered at the given dose by intragastric gavage at a volume of 1 mL/100 g, once daily for 10 consecutive days. One hour after the last administration, except for the Sham, The other groups underwent myocardial infarction model establishment by ligating the left anterior descending coronary artery (LADL) [39]. Briefly, rats were anesthetized by intraperitoneal injection of Urethane, 20% (0.5 mL/100 g), then intubated and ventilated with a ventilator (ventilator model HX-100E, Taimeng Biotechnology Co., Ltd., Chengdu, China). After opening the thoracic cavity, the pericardium was separated, and the left coronary artery was ligated with 6-inch sterile suture. The depth and width of the ligation were both 2 mm. The operation process for the sham operation group was the same, but only the suture was inserted without ligation.

### 4.3. H9c2 Cell Culture

H9c2 rat cardiomyocytes were purchased from the Cell Bank of the Chinese Academy of Sciences in Shanghai. This cell adheres to the surface and has a myoblast-like morphology. It is cultured in DMEM high-glucose medium supplemented with 10% fetal bovine serum and 1% penicillin-streptomycin, and maintained in a 37 °C, 5% CO_2_ and saturated humidity incubator under routine conditions.

### 4.4. Serum Biochemical Index Detection

24 h after the establishment of the SD rat model, blood was collected from the abdominal aorta and centrifuged at 3000 RPM for 20 min at 4 °C to obtain serum. Levels of lactate dehydrogenase (LDH), creatine kinase isoenzymes (CK-MB), malondialdehyde (MDA), and superoxide dismutase (SOD) were determined using commercial kits and microplate readers purchased from Nanjing Jiancheng Biotechnology Research Institute (Nanjing, China).

### 4.5. Tissue Index Detection

Heart tissues were collected and homogenized. ATP content was measured using an ATP assay kit (Beyotime Biotechnology, Shanghai, China) following the protocol provided. The activities of mitochondrial respiratory chain complexes I and IV were assessed using respective ELISA kits (Shanghai Jianglai Biotechnology Co., Ltd., Shanghai, China).

### 4.6. 2,3,5-Triphenyl-4-Thiazoline Chloride (TTC) Staining

Hearts were sectioned and stained with TTC to evaluate infarct size. Images were analyzed using Image-Pro Plus 6.0 software.

### 4.7. Target Identification via Photoaffinity Probe-Based Pull-Down Assays

The H9c2 cell lysate was prepared by ultrasonic treatment and centrifugation, and the protein concentration was determined by the Bradford method and adjusted to 2 mg/mL. For the M2 group, ISOV-functionalized magnetic beads (loading capacity: 0.57 μmol/mL) were used. The AC group was processed under the same conditions using control beads (without ISOV conjugation). The magnetic beads were pre-treated and subjected to three-level affinity enrichment (4 °C, 1 h per stage). The enriched proteins were purified, dissolved, enzymatically digested, and the peptide segments were desalted. They were labeled with TMT18plex, mixed in equal amounts, fractionally freeze-dried, and finally identified by liquid chromatography-tandem mass spectrometry (LC-MS/MS) (Q-Exactive HF-X, Thermo Fisher Scientific, Waltham, MA, USA).

### 4.8. Bioinformatics Analysis for Potential Targets of ISOV Against Myocardial Ischemia

The R language packages “clusterProfiler”, “org.Hs.eg.db”, “topGO”, and “DOSE” were used for gene ontology (GO) enrichment analysis.

### 4.9. Molecular Docking of ISOV with SLC25A4

The structure of ISOV was downloaded from the PubChem database (https://pubchem.ncbi.nlm.nih.gov/), and the structure was minimized using the Chem3D software (PerkinElmer, Inc., Waltham, MA, USA). The three-dimensional structure of SLC25A4 was obtained from the RCSB PDB database (https://www1.rcsb.org/). Water molecules and hydrogen atoms were added to the protein using the Discovery Studio Client (BIOVIA, San Diego, CA, USA). AutoDock Tools (The Scripps Research Institute, La Jolla, CA, USA), AutoDock Vina (The Scripps Research Institute, La Jolla, CA, USA), and Pyrx-0.8 (The Scripps Research Institute, La Jolla, CA, USA) were used to dock ISOV onto the SLC25A4 structure. The docking results were analyzed and visualized using Pymol (Schrödinger, Inc., New York, NY, USA).

### 4.10. In Situ Pull-Down Experiment

After H9c2 cells were thawed on ice, they were lysed with lysis buffer and sonicated. The supernatant was obtained after centrifugation at 4 °C for 30 mins at 20,000 RPM. The protein concentration was determined using the Bradford method and adjusted to 2 mg/mL. The functionalized ISOV magnetic beads and the blank control magnetic beads were washed rigorously, and then incubated with 2 mg of protein lysate in two stages (4 °C shaking, 50 μL of magnetic beads each time, with an interval of 1 h). After gradient washing of the magnetic beads in the pull-down experiment, the high-purity target complex was eluted with a strong reducing loading buffer (95 °C for 20 min) Sodium Dodecyl Sulfate-Polyacrylamide Gel Electrophoresis (SDS-PAGE) showed that the characteristic bands had molecular weights consistent with expectations). The elution products were subjected to immunoblotting analysis.

### 4.11. CETSA

After H9c2 cells were thawed on ice, they were lysed with lysis buffer and sonicated. The supernatant was obtained after centrifugation at 4 °C for 30 min at 20,000 RPM. The protein concentration was determined using the Bradford method and adjusted to 2 mg/mL. 450 μL of protein samples were mixed with DMSO or ISOV (100 μM), and incubated at 25 °C in the dark for 1 h. The samples were then aliquoted into 8 portions and incubated at the preset temperature gradients (25, 37, 42, 46, 50, 54, 58, 62, 67 °C) for 3 min, and the reaction was terminated by rapid ice bath. The supernatant was collected for immunoblotting analysis.

### 4.12. Western Blotting

Total protein extracted from H9c2 cells was separated by SDS-PAGE and transferred onto a polyvinylidene fluoride (PVDF) membrane. After blocking with 5% non-fat skimmed milk for 60 min, the membrane was incubated overnight at 4 °C with primary antibodies (all diluted 1:1000), followed by incubation with the corresponding horseradish peroxidase (HRP)-conjugated secondary antibody (diluted 1:1000) for 60 min. Antibody binding was detected using enhanced chemiluminescence, and band intensities were quantified using ImageJ software (version 1.54) (National Institutes of Health, Bethesda, MD, USA ). Primary antibodies used in this study targeted SLC25A4, AMPKα, p-AMPK, and PGC-1α (all rabbit-derived), with β-actin (also rabbit-derived) serving as the loading control. The secondary antibody was HRP-conjugated goat anti-rabbit IgG. The p-AMPK/AMPKα1 ratio was calculated by dividing the p-AMPK/β-actin value by the AMPKα1/β-actin value after densitometric quantification.

### 4.13. Lentivirus Interference and Transfection

The shRNA sequence targeting SLC25A4 (sequence and design details provided in Supplementary Materials, Section S2) was designed and synthesized by Chongqing Weisiteng Biomedical Technology Co., Ltd. (Chongqing, China). Sequence alignment confirming its specificity are presented in Supplementary Figures S1–S4. H9c2 cells were divided into four treatment groups: sh-NC (normoxia), sh-NC (hypoxia), sh-SLC25A4 (hypoxia), and sh-SLC25A4 (hypoxia) + ISOV.

For lentiviral infection, H9c2 cells were seeded in 6-well plates at a density of 0.5 × $10^{5}$ cells/mL and cultured until 70% confluence. The medium was then replaced with fresh medium containing 5 μg/mL polybrene. Lentiviral particles were thawed on ice and added at an multiplicity of infection (MOI) of 20. After 24 h of incubation, the virus-containing medium was replaced with fresh complete medium. To establish stable cell lines, transduced cells were selected with 1 μg/mL puromycin—a concentration predetermined to be the minimal lethal dose in wild-type H9c2 cells. Selection was maintained over three passages with puromycin-containing medium refreshed every two days.

### 4.14. CCK8 Assay

The effects of ISOV on H9c2 cell viability under both normoxic and hypoxic conditions have been previously evaluated using multiple concentrations (2.5, 5, 10, 20, and 40 μM), as described in our earlier publication [14]. Stably transduced H9c2 cells were seeded into 96-well plates at 8 × $10^{3}$ cells per well in DMEM supplemented with 10% FBS. At 60–80% confluence, the sh-SLC25A4 + ISOV group was pretreated with 40 μM ISOV for 8 h, while the other groups received corresponding treatments. All groups except the sh-NC (normoxia) group were subjected to hypoxia (1% O_2_, 5% CO_2_, 94% N_2_) for 1 h. Then, 10 μL of CCK-8 solution was added to each well and incubated for 1–4 h. Absorbance was measured at 450 nm using a microplate reader.

### 4.15. ATP Level Detection

Total proteins were extracted from each group of H9c2 cells, and the ATP concentration was determined using an enhanced ATP detection kit (Beyotime Biotechnology, Shanghai, China).

### 4.16. Flow Cytometry Detection of MMP

Total proteins were extracted from each group of H9c2 cells, and the MMP was measured using the JC-10 apoptosis MMP detection kit (Beyotime Biotechnology, Shanghai, China) on a flow cytometer (CytoFLEX, Beckman Coulter, Brea, CA, USA).

### 4.17. Flow Cytometry Detection of the Openness of mPTP

Total proteins were extracted from each group of H9c2 cells, and the mPTP was determined using the mPTP detection kit (Beyotime Biotechnology, Shanghai, China) on the same flow cytometer (Beckman Coulter).

### 4.18. Statistical Analysis

All data are presented as the mean ± standard error of the mean (SEM). For animal experiments, n = 8; for cell experiments, n = 3. Statistical analysis was performed using GraphPad Prism 10.0. Unless otherwise stated, comparisons among multiple groups were evaluated using one-way ANOVA, while comparisons between two groups were assessed using Student’s *t*-test. A *p*-value < 0.05 was considered statistically significant.

## 5. Conclusions

**Figure 6:**
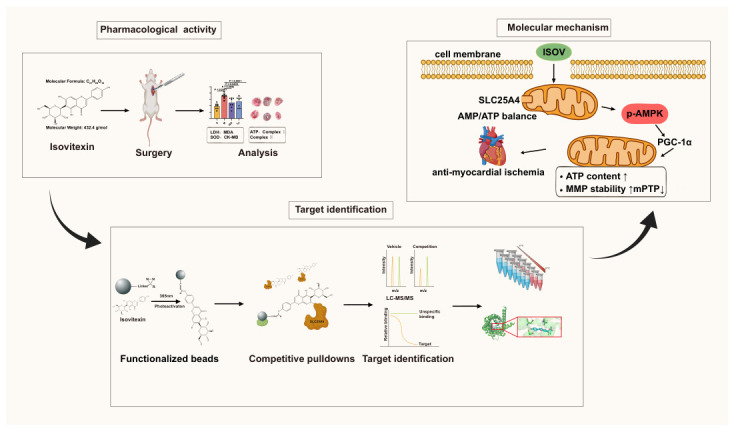
ISOV ameliorates post-ischemic myocardial injury by targeting SLC25A4 and modulating the AMPK/PGC-1α signaling pathway.

This study demonstrates that ISOV, the active component of Tsantan Sumtang San, exerts anti-myocardial ischemic effects by directly targeting the mitochondrial ADP/ATP translocase SLC25A4. Binding of ISOV to SLC25A4 restores mitochondrial ADP/ATP exchange efficiency, thereby alleviating energy depletion, stabilizing the mitochondrial membrane potential, and inhibiting mPTP opening. Moreover, ISOV enhances the coordinated activation of the AMPK/PGC-1α signaling axis, promotes mitochondrial biogenesis, and improves cellular energy homeostasis (Figure 6). These findings not only elucidate the mechanistic basis of Tsantan Sumtang San from a modern pharmacological perspective but also provide a theoretical rationale for developing novel anti-ischemic agents targeting the SLC25A4-AMPK/PGC-1α pathway. A limitation of this study is that the precise regulatory relationship between SLC25A4 and AMPK/PGC-1α signaling remains incompletely defined, warranting further investigation.

## Data Availability

The original contributions presented in this study are included in the article/Supplementary Material. Further inquiries can be directed to the corresponding author(s).

## Supplementary Materials

The following supporting information can be downloaded at: https://www.mdpi.com/article/10.3390/ijms27104193/s1.

## Abbreviations

The following abbreviations are used in this manuscript:

ISOV	Isovitexin
LADL	Ligation of the left anterior descending branch of the coronary artery
CETSA	Cellular thermal shift assay
LDH	Dehydrogenase
CK-MB	Creatine kinase isoenzymes
MDA	Malondialdehyde
SOD	Superoxide dismutase
AMPK	Adenosine monophosphate (AMP)–activated protein kinase
PGC1-α	Proliferator-activated receptor gamma coactivator-1α
MMP	Mitochondrial membrane potential
mPTP	Mitochondrial Permeability Transition Pore
TTC	2,3,5-Triphenyl-4-thiazoline Chloride
GO	Gene ontology
SDS-PAGE	Sodium Dodecyl Sulfate-Polyacrylamide Gel Electrophoresis
PVDF	Polyvinylidene fluoride
LC-MS/MS	Liquid chromatography-tandem mass spectrometry
