# Supplementary material for: Isovitexin Alleviates Myocardial Ischemia by Targeting SLC25A4 and Modulating the AMPK/PGC-1α Signaling Pathway
Source: Int J Mol Sci. 2026 May 8;27(10):4193. doi: 10.3390/ijms27104193 (PMC13206432; doi:10.3390/ijms27104193)
Supplement: Supplementary file 1 [file ijms-27-04193-s001.zip › Supplementary_TableS1.pdf]

Table S1 Chemicals and reagents

Category	Reagent / Kit	Supplier / Brand
**Buffers & Basic Chemicals**	PBS Powder	Sigma
	DPBS (Dulbecco's Phosphate Buffered Saline)	ThermoFisher Scientific
	Tris	Siyareagent
	Glycine	Sangon Biotech
	SDS	BBI
	DTT (DL-Dithiothreitol, 99%)	Innochem
	Iodoacetamide	Sigma-Aldrich
	Formic acid	Fluka
	Hydroxylamine solution	Aldrich
	TMT 18plex	Thermo
	DEPC-treated Water	Sangon Biotech
	Absolute Ethanol (AR Grade)	Haixing
	Methanol	Sinopharm Chemical Reagent
	DMSO	Sigma
**Cell Culture & Transfection**	DMEM Medium	Gibco
	Fetal Bovine Serum (FBS)	Gibco
	Trypsin	Beyotime
	Penicillin-Streptomycin Solution (100X)	Beyotime

Category	Reagent / Kit	Supplier / Brand
	Opti-MEM	Gibco
	DH5α Competent Cells	TransGen Biotech
	Plasmid Miniprep Kit	Omega
	Plasmid Maxiprep Kit	Omega
	AgeI	TransGen Biotech
	EcoRI	TransGen Biotech
**Compounds**	Isovitexin	MACKLIN
	Methyl Ferulate	MACKLIN
	Trimetazidine Dihydrochloride	MACKLIN
**Detection Kits (Cell/Biochemistry)**	LDH Assay Kit	NJJC
	SOD Assay Kit	NJJC
	MDA Assay Kit	NJJC
	CK-MB ELISA Kit	JL
	ATP Assay Kit	JL / Beyotime
	Rat Mitochondrial Complex I ELISA Kit	JL
	Rat Mitochondrial Complex IV ELISA Kit	JL
	MPTP Assay Kit	Beyotime

Category	Reagent / Kit	Supplier / Brand
	Detergent Compatible Bradford Protein Assay Kit	Beyotime
	TaKaRa BCA Protein Assay Kit	Takara
	BCA Protein Quantification Kit	YEASEN
	CCK-8 Kit	Beyotime
	Cell Apoptosis & Mitochondrial Membrane Potential Assay Kit (JC-10)	KeyGEN BioTECH
**Molecular Biology**	TRIeasy™ Total RNA Extraction Reagent	YEASEN
	RNA Extraction Buffer	YEASEN
	Hifair® AdvanceFast 1st Strand cDNA Synthesis SuperMix	YEASEN
	Hieff UNICON® qPCR SYBR Green Master Mix	YEASEN
	Hifair® III 1st Strand cDNA Synthesis Kit	YEASEN
	Hieff UNICON® Universal Blue qPCR SYBR Green Master Mix	YEASEN
	RNAiso plus	Takara
	Primers	Youkang Biology
**Proteomics**	rLys-C, MS Grade	EnzySource
	rAc-Trypsin, MS Grade	EnzySource

Category	Reagent / Kit	Supplier / Brand
**Western Blot**	Antibody: SLC25A4 (Rabbit)	Nature Bioscience
	Antibody: AMPKα1 (Rabbit)	Boster
	Antibody: p-AMPK (Rabbit)	Affinity
	Antibody: PGC1-α (Rabbit)	Boster
	Antibody: β-actin (Rabbit)	HuaAn
	Secondary Antibody: HRP-conjugated Goat Anti-Rabbit IgG	Beyotime
	SDS-PAGE Protein Loading Buffer (5X)	Beyotime
	TEMED	Amersham Pharmacia Biotech
	180 kDa Prestained Protein Marker	YEASEN
	RIPA Lysis Buffer	Beyotime
	Skim Milk Powder	Yili
	Filter Paper	Whatman
	30% Acrylamide Solution	Maokang Biology
**Stains & Dyes**	2,3,5-Triphenyltetrazolium Chloride (TTC) Stain	SOLARBIO
