# Supplementary material for: Isovitexin Alleviates Myocardial Ischemia by Targeting SLC25A4 and Modulating the AMPK/PGC-1α Signaling Pathway
Source: Int J Mol Sci. 2026 May 8;27(10):4193. doi: 10.3390/ijms27104193 (PMC13206432; doi:10.3390/ijms27104193)
Supplement: Supplementary file 1 [file ijms-27-04193-s001.zip › Supplementary_SectionS2.pdf]

Section S2 shRNA Sequences and Cloning Information

Slc25a4-shRNA1：

Sense：ccggggatcaggctttgagcttcctctcgagaggaagctcaaagcctgatcctttttg

Antisense：aattcaaaaaggatcaggctttgagcttcctctcgagaggaagctcaaagcctgatcc

Slc25a4-shRNA2：

Sense：ccggggtcaaactgctgctgcaggtctcgagacctgcagcagcagtttgacctttttg

Antisense：aattcaaaaaggtcaaactgctgctgcaggtctcgagacctgcagcagcagtttgacc

Slc25a4-shRNA3：

Sense：ccggggacagttgactgctggaggactcgagtcctccagcagtcaactgtcctttttg

Antisense：aattcaaaaaggacagttgactgctggaggactcgagtcctccagcagtcaactgtcc

NC-shRNA：

Sense：ccggcctaaggttaagtcgccctcgctcgagcgagggcgacttaaccttaggtttttg

Antisense：aattcaaaaacctaaggttaagtcgccctcgctcgagcgagggcgacttaaccttagg

Figure Legend

Fig. S1-S4. Sequence alignment confirming probe specificity.
