# Supplementary figures and images for: Isovitexin Alleviates Myocardial Ischemia by Targeting SLC25A4 and Modulating the AMPK/PGC-1α Signaling Pathway
Source: Int J Mol Sci. 2026 May 8;27(10):4193. doi: 10.3390/ijms27104193 (PMC13206432; doi:10.3390/ijms27104193)

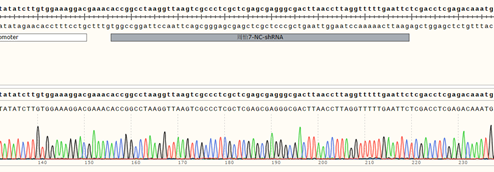

Supplement: Supplementary file 1 [file ijms-27-04193-s001.zip › FigS4.tif]

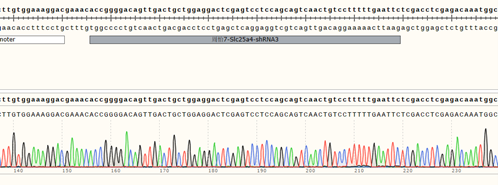

Supplement: Supplementary file 1 [file ijms-27-04193-s001.zip › FigS3.tif]

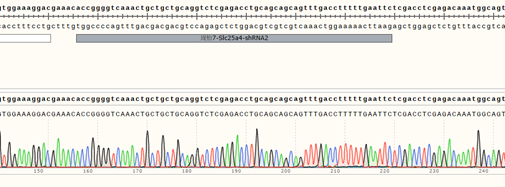

Supplement: Supplementary file 1 [file ijms-27-04193-s001.zip › FigS2.tif]

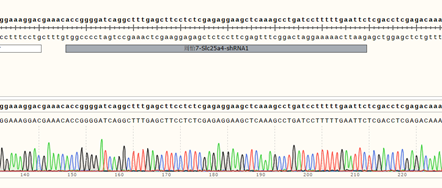

Supplement: Supplementary file 1 [file ijms-27-04193-s001.zip › FigS1.tif]
